# Comprehensive beam delivery latency evaluation for gated proton therapy system using customized multi‐channel signal acquisition platform

**DOI:** 10.1002/acm2.14349

**Published:** 2024-03-29

**Authors:** Zhipeng Liu, Lingjing Pan, Tao Ma, Hsiao‐Ming Lu, Yuanyuan Wang

**Affiliations:** ^1^ Hefei Ion Medical Center the First Affiliated Hospital of USTC Division of Life Sciences and Medicine University of Science and Technology of China Hefei China; ^2^ Ion Medical Research Institute University of Science and Technology of China Hefei China

**Keywords:** gating, latency, ProBeam, RPM

## Abstract

**Purpose:**

Beam delivery latency in respiratory‐gated particle therapy systems is a crucial issue to dose delivery accuracy. The aim of this study is to develop a multi‐channel signal acquisition platform for investigating gating latencies occurring within RPM respiratory gating system (Varian, USA) and ProBeam proton treatment system (Varian, USA) individually.

**Methods:**

The multi‐channel signal acquisition platform consisted of several electronic components, including a string position sensor for target motion detection, a photodiode for proton beam sensing, an interfacing board for accessing the trigger signal between the respiratory gating system and the proton treatment system, a signal acquisition device for sampling and synchronizing signals from the aforementioned components, and a laptop for controlling the signal acquisition device and data storage. RPM system latencies were determined by comparing the expected gating phases extracted from the motion signal with the trigger signal's state turning points. ProBeam system latencies were assessed by comparing the state turning points of the trigger signal with the beam signal. The total beam delivery latencies were calculated as the sum of delays in the respiratory gating system and the cyclotron proton treatment system. During latency measurements, simulated sinusoidal motion were applied at different amplitudes and periods for complete beam delivery latency evaluation under different breathing patterns. Each breathing pattern was repeated 30 times for statistical analysis.

**Results:**

The measured gating ON/OFF latencies in the RPM system were found to be 104.20 ± 13.64 ms and 113.60 ± 14.98 ms, respectively. The measured gating ON/OFF delays in the ProBeam system were 108.29 ± 0.85 ms and 1.20 ± 0.04 ms, respectively. The total beam ON/OFF latencies were determined to be 212.50 ± 13.64 ms and 114.80 ± 14.98 ms.

**Conclusion:**

With the developed multi‐channel signal acquisition platform, it was able to investigate the gating lags happened in both the respiratory gating system and the proton treatment system. The resolution of the platform is enough to distinguish the delays at the millisecond time level. Both the respiratory gating system and the proton treatment system made contributions to gating latency. Both systems contributed nearly equally to the total beam ON latency, with approximately 100 ms. In contrast, the respiratory gating system was the dominant contributor to the total beam OFF latency.

## 1 INTRODUCTION

Respiration‐induced motion in the thorax and abdomen is widely recognized challenge for radiotherapy, often resulting in unexpected side effects on healthy tissue surrounding the tumor target.^1^, ^2^, ^3^ The motion not only leads to the variation in tumors location but also alters tissue density along the beam path, potentially compromising the depth‐dose distribution in particle therapy.^4^, ^5^, ^6^ To address these issues, various motion management techniques, such as gating and breath‐holding, have been proposed. These techniques aim to optimize target dose delivery while minimizing exposure to healthy tissue by restricting beam delivery to specific phases or displacement ranges.^7^ Whenever the observed surrogate, such as an infrared reflecting marker block or selected region of interest from optical surface monitoring system, enters or exits a pre‐set gating window, the treatment system should turn on or turn off the beam immediately to prevent off‐target scenarios. However, in practice, beam delivery latencies cannot be completely eliminated. Respiratory gating systems require time to process signals acquired from sensors and generate beam ON/OFF trigger signals for treatment systems like LINACs or cyclotrons. Treatment systems also need time to respond the trigger signals with the appropriate operations to turn on/off the beam. These above‐mentioned delays from both respiratory gating systems and treatment systems collectively impact the dose delivery accuracy. Therefore, the American Association of Physicists in Medicine (AAPM) task group 142 (TG‐142) and task group 198 (TG‐198) have recommended that for LINAC‐based radiotherapy when the object's speed is less than 20 mm/s, the total gating latencies should be maintained below 100 ms.^8^, ^9^

In our proton therapy center, conducting a comprehensive evaluation of the end‐to‐end gating latencies in various clinical scenarios is an essential component of our commissioning process for the gated proton treatment system. Since both the respiratory gating system and proton treatment system (i.e., cyclotron system) contribute to beam delivery latencies, it is essential to comprehend the latencies unique to each system. It helps physicists and radiotherapy vendors to optimize the beam control process of the cyclotron or upgrade the existing motion management system.

Currently, there is neither commercially available quality assurance tool nor established guideline to verify gating latencies. Therefore, some specialists in the area of medical physics have developed individual solutions to measure the gating latencies.^10^, ^11^, ^12^, ^13^, ^14^, ^15^ All these solutions only focused on the end‐to‐end latency measurements with various gated treatment systems. The objective of this work was to design a reliable multi‐channel signal acquisition platform to measure the respiratory gating system delays and the proton treatment system delays separately under different simulated breathing patterns. This platform serves as a useful tool to perform periodic latency check on the gated proton system.

## 2 MATERIAL AND METHODS

**FIGURE 1:**
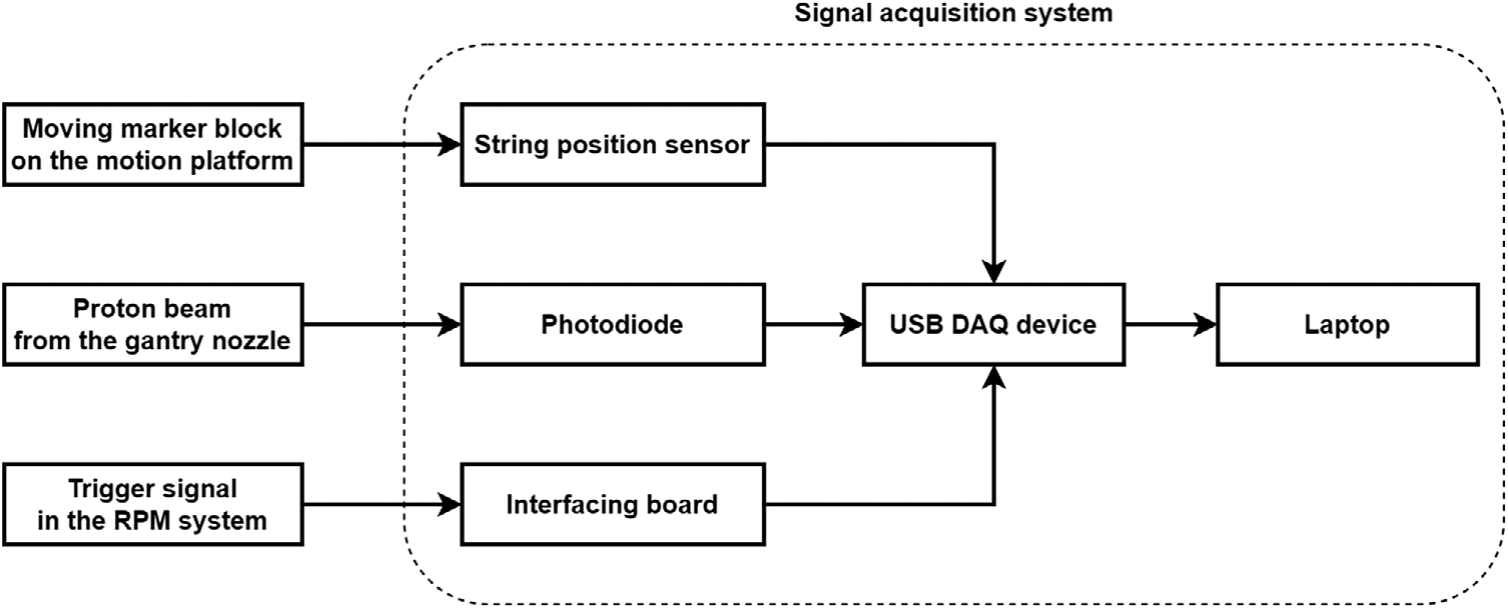
Schematic diagram of the signal acquisition platform.

Latency measurements were performed at our ProBeam spot scanning proton therapy system which is integrated with the RPM (Real‐time Position Management) respiratory gating system (version 1.8). Both systems controlled the gating process, commencing with motion monitoring by the RPM system. Real‐time processing of images captured by an infrared camera enabled the extraction of the position of the infrared reflecting marker block. When the computer recognized that the target entered or exited the predefined gating window, it triggered an immediate transition in the state of the trigger signal (RPM output signal) between HIGH and LOW. The proton system received trigger signal and started the corresponding mechanism to turn on/off the beam. To sense the target position, the trigger state, and the beam state at same time, a three‐channel real‐time signal acquisition platform (shown in Figure 1) was designed in this work. This platform consists of several electronic components, including a string position sensor (Model MPS‐XS, Miran Technology, China) to detect mimic respiratory motion, aphotodiode with 10 × 10 mm^2^ sensing area (Model LSSPD‐10, Beijing Light Sensing Technologies, China) for detecting proton beams, an interfacing board to access the trigger signal generated by the RPM system, an electrical signal acquisition device (USB‐6009, National Instruments, USA) to sample and synchronize signals from different channels, and a laptop for controlling the platform and recording data.

**FIGURE 2:**
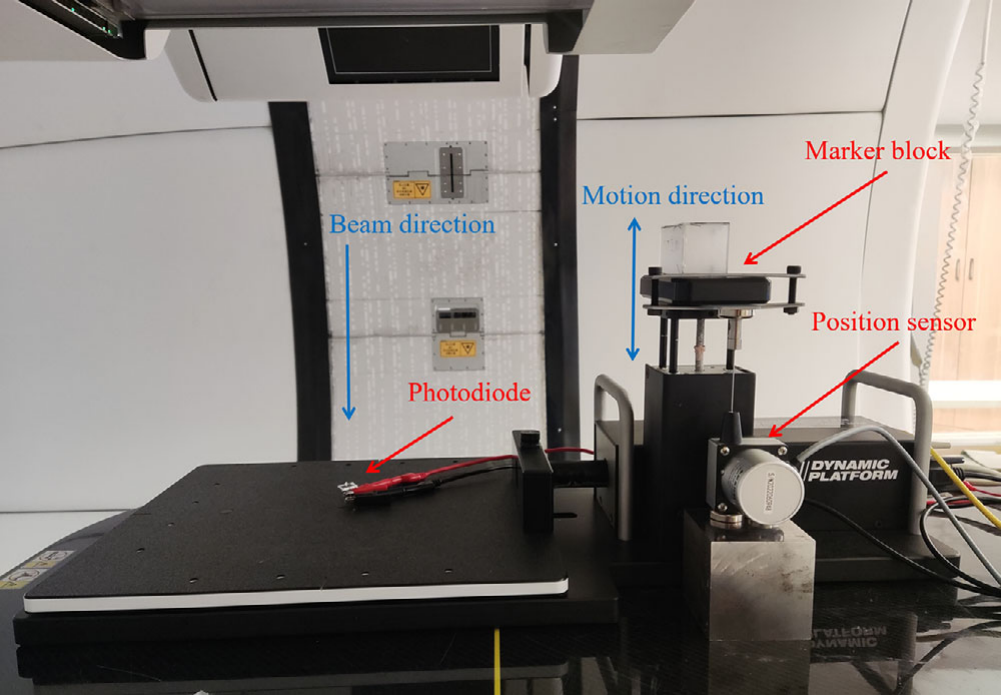
Deployment of the signal acquisition platform in the proton treatment room.

As illustrated in Figure 2, the modified platform was deployed on the couch in the proton treatment room. An infrared reflecting marker block was positioned on the surrogate moving stage of a commercial motion simulator (Model 008PL, CIRS, USA). Whenever the simulator executed vertical surrogate movements, the RPM system detected the motion in real‐time via an on‐ceiling fixed infrared camera operating at 25 fps. For monitoring the vertical motion of the marker block, a string position sensor was utilized. The rear side of the sensor was attached to an iron block near the motion simulator using a magnet, and the front detection head of the sensor was linked to the surrogate stage of the motion simulator by a customized metal structure. The sensor had a detection range of up to 100 mm, and the corresponding analog voltage output extended up to 10 V. The sensor linearity was better than 0.05% F.S. and the repeatability was within 0.02% F.S. The photodiode was mounted on the central plate of the motion simulator and covered by aluminum folie to avoid interference from ambient light. Proton beam irradiation could induce an increase in voltage of around 100 millivolts in the diode. The trigger signal could be accessed through the interfacing board in the control box of the RPM system. It featured only two states: 0 V indicated beam OFF and 12 V indicated beam ON. Following the setup process, the position sensor, the photodiode and the interfacing board were connected to the USB data acquisition device as three independent analog input channels. All signals from these different channels were able to be synchronized and recorded on the laptop. Subsequently, the photodiode was aligned with the isocenter for proton beam detection. A gated proton treatment plan with an additional range shifter was designed to realize monoenergetic single spot beam delivery. The spot size at isocenter was approximately 20 mm, effectively covering the entire sensing area of the photodiode. To investigate the gating delays in different treatment scenarios, the motion simulator was programmed to perform vertical sinusoidal motion with periods of 3, 4, 5 s, and the amplitude was set from 5 to 20 mm with 5 mm step length. In each motion scenario, the gating window was fixed at 40%−60%, and the measurement performed 30 cycles at 10 kHz sampling rate for further statistical analysis.

An in‐house designed software was developed in LabVIEW for three‐channel data analysis. The analysis comprised several key steps. In the first step, a notch filter and moving median filter were used to reduce the internal system noise in the position sensor signal. Following noise suppression, the second step was gating phase localization which is similar to the procedure Wiersma et al.^15^ proposed. In this step, a threshold was defined as 90% peak to peak motion amplitude to isolate peak region for each sinusoidal motion cycle. Then each induvial peak region was fitted to a polynomial function by using least square method. The positions of maximal values from these fitted curves were then extracted and used as reference points to calculate the expected beam‐on phase, falling within the 40%–60% phase interval, for each motion cycle. The third step was to calculate RPM system delays and ProBeam system delays separately. This was accomplished by distinguishing between beam ON/OFF regions and RPM gating ON/OFF regions through the definition of HIGH and LOW signal states. For both the beam signal and the trigger signal, the raising edge and falling edge were set to 20% and 80% of its maximal value. In the final step, the RPM system gating delays was determined by the temporal comparison of the expected gating phases extracted from the motion signal and the state turning points of trigger signal, as described in Equations (1) and (2).

(1) $$\tau_{RPM-on}\;=\;T_{trigger-on}\;-\;T_{phase-40\%}$$

(2) $$\tau_{RPM-off}\;=\;T_{trigger-off}\;-\;T_{phase-60\%}$$

The RPM system gating ON latency (*τ*_RPM‐on_) was defined as the time delay from the marker block entered the gating window that is, 40% phase position (*T*_phase‐40%_) according to the actual trigger signal switched from OFF to ON (*T*_trigger‐on_). Similarly, the RPM system gating OFF latency (*τ*_RPM‐off_) was defined as the duration from when the block exited the gating window, at the 60% phase position (*T*_phase‐60%_), to the instant when the actual trigger signal state switched from ON to OFF (*T*_trigger‐off_).

The ProBeam system gating latencies, that is, the cyclotron gating ON latency (*τ*_CYC‐on_) and the cyclotron gating OFF latency (*τ*_CYC‐off_) were determined by comparing the state turning points of the trigger signal and the beam signal, as described in Equations (3) and (4).

(3) $$\tau_{CYC-on}\;=\;T_{beam-on}\;-\;T_{trigger-on}$$

(4) $$\tau_{CYC-off}\;=\;T_{beam-off}\;-\;T_{trigger-off}$$

The cyclotron gating ON latency described the time difference between the state of the trigger signal switched from OFF to ON (*T*_trigger‐on_) and beam ON signal detected by the photodiode (*T*_beam‐on_). Similarly, the cyclotron gating OFF latency was defined as the time delay from the trigger OFF (*T*_trigger‐off_) to beam OFF (*T*_beam‐off_).

The total beam OFF delivery latency (*τ*_total‐on_) and total beam OFF delivery latency (*τ*_total‐off_) were finally calculated based on Equations (5) and (6) which were simply derived from Equations (1), (3) and Equations (2), (4).

(5) $$\begin{array}{ccc} \tau_{total-on}\; & = & \;\tau_{RPM-on\;}+\;\tau_{CYC-on}\\ & = & \;T_{beam-on}\;-\;T_{phase-40\%} \end{array}$$

(6) $$\begin{array}{ccc} \tau_{total-off} & = & \tau_{RPM-off}\;\;+\;\tau_{CYC-off}\\ & = & \;T_{beam-off}\;-\;T_{phase-60\%} \end{array}$$

## 3 RESULTS

**FIGURE 3:**
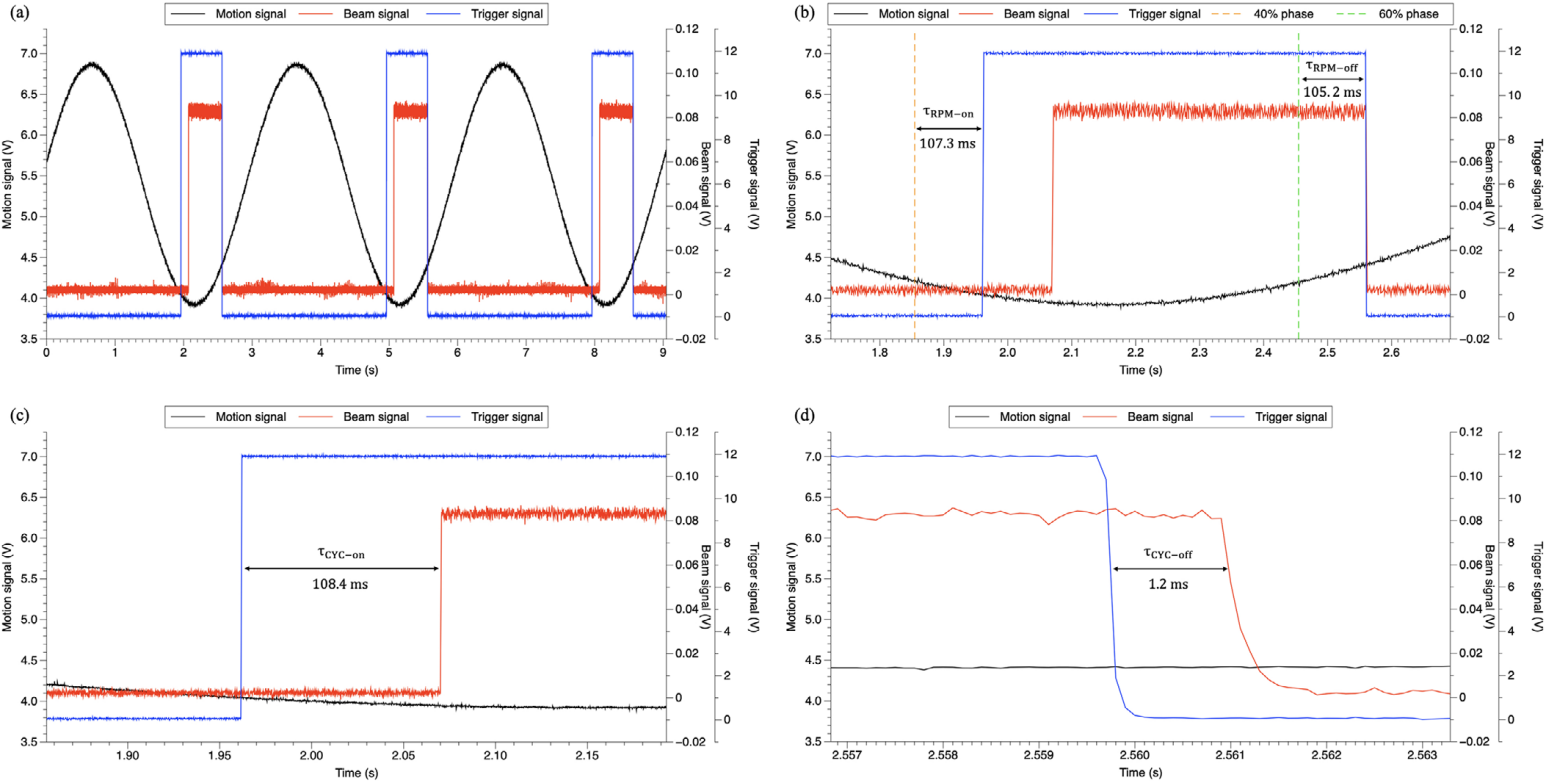
Example raw data of the three‐channel signals.

Figure 3 shows an sample of recorded raw data which describes a repeated real gating process in which the motion simulator was programed to perform repeatedly sinusoidal motion with an amplitude of 15 mm and a period of 3 s. Figure 3(a) displays a 9 s segment of the synchronized signals obtained from the position sensor (black lines), the photodiode (red lines), and interfacing board (blue lines). Figure 3(b) presents the magnified region which includes a complete gating process controlled by RMP system and the ProBeam system. Orange/green dashed lines mark the expected gate ON/OFF times (40%, 60% phase) based on calculations from our in‐house designed software. This illustrates that RPM gating latencies between the expected gate ON/OFF times and the trigger ON/OFF times are approximately 100 ms. Figure 3(c) and (d) provide the further zoomed in regions to illustrate cyclotron gating delays in the beam ON/OFF switching process. it took 108.4 and 1.2 ms, respectively, from receiving the trigger ON/OFF signals to delivering/stopping the proton beam. In Figure 3(d), it can be overserved that, the temporal resolution of the multi‐channel signal acquisition system is enough to detect the lag times on the millisecond time scale.

The comprehensive results of the complete beam latency investigation across 11 motion patterns are summarized in Table 1. The statistics are calculated based on the motion cycles which were repeated 30 times for each motion pattern. In various respiratory motion scenarios, the RPM gating ON latency varied from 82.19 to 113.99 ms, and the RPM gating OFF latency was in the range between 102.34 and 124.95 ms. Conversely, the measured cyclotron gating ON/OFF remained consistently at around 108 and 1.2 ms, respectively.

**TABLE 1:** Beam delivery latencies under different motion scenarios.

Amplitude (mm)	Period (s)	*τ*_RPM‐on_ (ms)	*τ*_RPM‐off_ (ms)	*τ*_CYC‐on_ (ms)	*τ*_CYC‐off_ (ms)	*τ*_total‐on_ (ms)	*τ*_total‐off_ (ms)
5	3	108.31 ± 15.02	112.53 ± 1.06	108.21 ± 0.67	1.20 ± 0.03	216.52 ± 15.02	113.73 ± 1.07
	4	105.26 ± 4.46	102.34 ± 1.80	108.12 ± 0.69	1.20 ± 0	213.38 ± 4.45	103.54 ± 1.80
	5	82.19 ± 18.24	103.91 ± 2.51	108.45 ± 0.75	1.20 ± 0.04	190.64 ± 18.20	105.11 ± 2.51
10	3	112.52 ± 18.58	124.94 ± 2.61	108.54 ± 0.74	1.20 ± 0.02	221.05 ± 18.51	126.15 ± 2.61
	4	101.60 ± 3.18	107.94 ± 15.96	108.26 ± 0.86	1.20 ± 0.04	209.86 ± 3.26	109.13 ± 15.97
	5	104.96 ± 7.62	111.10 ± 16.39	108.50 ± 0.99	1.19 ± 0.03	213.46 ± 7.73	112.28 ± 16.39
15	3	106.02 ± 2.91	111.00 ± 15.56	108.60 ± 1.38	1.19 ± 0.04	214.62 ± 3.31	112.19 ± 15.55
	4	113.99 ± 18.28	124.95 ± 12.47	108.18 ± 0.70	1.20 ± 0.02	222.17 ± 18.29	126.15 ± 12.47
	5	103.03 ± 4.09	124.09 ± 18.39	108.06 ± 0.62	1.19 ± 0.04	211.08 ± 4.12	125.28 ± 18.38
20	4	105.58 ± 2.83	107.08 ± 11.28	108.22 ± 0.80	1.19 ± 0.04	213.79 ± 3.04	108.28 ± 11.29
	5	102.80 ± 2.09	119.71 ± 18.72	108.11 ± 0.64	1.21 ± 0.05	210.91 ± 2.20	120.92 ± 18.72
Mean		104.20 ± 13.64	113.60 ± 14.98	108.29 ± 0.85	1.20 ± 0.04	212.50 ± 13.64	114.80 ± 14.98

## 4 DISCUSSIONS

Reviewing the reported gating latency measurements, the most widely used method was based on radiographic film. In this method, dose profiles were extracted from the exposure areas of the film, and gating delays were estimated based on the measured and expected dose profile lengths.^10^, ^11^, ^12^ While this indirect method is convenient to implement but susceptible to induce errors. For specific LINACs, the MV imaging system based method was employed. Barfield et al. used an electronic portal imaging device (EPID) to detect photon beams and moving phantom position in real‐time. Total gating delays were estimated by comparing the actual beam ON/OFF times with the expected ones.^13^ Nonetheless, the method was not suitable for facilities without EPID, such as proton or heavy ion treatment system, and its accuracy was limited by an imaging update period of 80 ms. Alternatively, Worm et al. employed a commercial camera and a scintillating crystal to measure the end‐to‐end gating latencies in proton system and LINAC system. The crystal worked as beam detector, emitting visible light when the photon or proton beam was active. Simultaneously, a camera operating at a 120 Hz sampling rate captured simulated vertical motions.^14^ The only disadvantage was its focus on total gating latency measurements, without the ability to separate the delays within the motion management system and the beam delivery system from the end‐to‐end latencies. In terms of measurement accuracy and compatibility, the method proposed by Wiersma et al. was tested across four different respiratory gating systems. In their approach, a linear potentiometer was used to convert phantom motions into voltage outputs, while a diode was applied to transform the photon beams into electrical signals. Both the motion signals and the beam signals could be collected and synchronized by a digital oscilloscope at the sampling rate of 2.5 kHz. Total beam lag times were estimated by temporally comparing the recorded signals in different oscilloscope channels.^15^ Nevertheless, the complex electrical circuit design work made the solution not easily accessible. Similar to the approach by Worm et al., independent gating delay measurements for both the respiratory gating system and treatment system were not feasible.^14^

Compared with the methods based on film, our approach was able to directly measure the gating latencies for each individual motion cycle. The multi‐channel capability allowed for the measurement of multiple delays, enabling the decoupling of the gating lags in the respiratory gating system and the proton treatment system from the end‐to‐end beam delivery latencies. This approach can be extended to other gated treatment systems and allows the individual delays from different components or systems to be decoupled from the total latency. The three‐channel signal acquisition platform we developed in this work was based on an existing commercial motion simulator. The total cost of modifications, which covered sensors, signal acquisition device, and metal structures, amounted to less than 5000 CNY (approximately 700 USD). By system component selection, commercial off‐the‐shelf products were chosen to minimize the need for electrical and electronic design work. The primary work for integration was to connect all the components with officially provided signal interfaces. The platform had a maximum sampling rate of 48 kHz, although we set it to 10 kHz for our specific application. With the high temporal resolution, it is possible to measure latencies on the millisecond time scale. The measurement errors induce by sampling time interval can be simply ignored. The accuracy of expected gating phase calculation was also checked in this work by visual examination of some random selected motion peak regions. The difference between manual selected peak position and the software calculated position was within 3 ms. The motion simulator and the signal acquisition system could be remotely controlled outside the treatment room where the platform deployed. The data analysis process was fully executed by the in house designed software. Hence, the total time consumption from measurement to data analysis was significantly reduced. The comprehensive latency evaluation we made in the 11 motion scenarios with total 330 motion cycles for each treatment room was less than 90 min.

Worm et al. investigated the proton beam delivery latency within the same gated proton treatment system (ProBeam + RPM version 1.75) using a scintillating crystal and a consumer‐grade camera.^14^ Their reported average latencies of *τ*_total‐on_ = 255 ± 23 ms and *τ*_total‐off_ = 95 ± 23 ms agree with our measured average latencies of *τ*_total‐on_ = 212.50 ± 13.64 ms and *τ*_total‐off_ = 114.80 ± 14.98 ms. Our *τ*_total‐on_ was approximately 40 ms smaller, which could be caused by RPM system upgrade (version 1.8 vs. version 1.75). As noted by Worm et al., differences in the beam ON/OFF switch mechanisms of the cyclotron result in a discrepancy between *τ*_total‐on_ and *τ*_total‐off_. The proton beam can be rapidly dumped using deflector plates, but it takes a relatively longer time to be re‐established.^14^ This conclusion was quantified by our cyclotron system gating delay results of *τ*_CYC‐off_ = 1.20 ± 0.04 ms and *τ*_CYC‐on_ = 108.29 ± 0.85 ms. According to the best of our knowledge, the cyclotron system gating latencies and their contributions to the total beam delivery latencies during gated proton treatments have not been previously reported in the published literature.

According to TG‐142 recommendations, the delay between actual gated beam and the expected gating window in photon radiation therapy should be less than 100 ms.^8^ Nevertheless, for proton radiation therapy, the official latency tolerance has not been released by any organization. Whether the latency tolerance used for photon radiation therapy applies to proton radiation therapy is still under investigation. In despite of the delay contributions from the RPM system, the cyclotron‐only gating ON delay of over 100 ms was much longer than expected. In contrast, the cyclotron gating OFF delay of approximately 1 ms was much better than expected. For patient safety, we concern more about the gating OFF lag times, because gating OFF latency corresponds to the time of beam delivering outside the gating window. At our facility, it is necessary to optimize the beam switch‐on process in the cyclotron.

## 5 CONCLUSIONS

In conclusion, we have successfully developed a three‐channel signal acquisition platform with high temporal resolution for measuring the gating latencies within our gated proton therapy system. With this decoupling method, it was able to investigate the gating lags happened in both of the respiratory gating system and the proton treatment system. The platform's resolution proved to be sufficient for distinguishing delays on the millisecond time scale. At our facility, both the RPM system and the ProBeam system played significant roles in contributing to gating latencies. In terms of the total beam ON latency, both systems made nearly equal contributions, amounting to approximately 100 ms. Conversely, for the total beam OFF latency, the RPM system played a significantly dominant role.

## AUTHOR CONTRIBUTIONS

Zhipeng Liu designed the signal acquisition platform, developed the gating latency analysis software, and drafted the manuscript. Lingjing Pan verified the signal acquisition platform and co‐drafted the manuscript. Tao Ma and Lingjing Pan were involved in measurement and data collection. Hsiao‐Ming Lu was responsible for reviewing the data and editing the manuscript. Yuanyuan Wang was responsible for study design and providing expertise on the direction and scope of the project. All author revised the manuscript and approved the submitted version.

## CONFLICT OF INTEREST STATEMENT

The authors have no related conflicts of interest to report.
